# RNA Sequencing Data Sets and Their Whole-Genome Sequence Assembly of Dengue Virus from Three Serial Passages in Vero Cells

**DOI:** 10.1128/MRA.00145-21

**Published:** 2021-04-29

**Authors:** Thidathip Wongsurawat, Nuntaya Punyadee, Piroon Jenjaroenpun, Dumrong Mairiang, Nattaya Tangthawornchaikul, Prida Malasit, Panisadee Avirutnan, Prapat Suriyaphol, Kwanrutai Chin-inmanu

**Affiliations:** a Division of Bioinformatics and Data Management for Research, Research Group and Research Network Division, Research Department, Faculty of Medicine Siriraj Hospital, Mahidol University, Bangkok, Thailand; b Division of Dengue Hemorrhagic Fever Research, Faculty of Medicine Siriraj Hospital, Mahidol University, Bangkok, Thailand; c Siriraj Center of Research Excellence in Dengue and Emerging Pathogens, Faculty of Medicine Siriraj Hospital, Mahidol University, Bangkok, Thailand; d Molecular Biology of Dengue and Flaviviruses Research Team, Medical Molecular Biotechnology Research Group, National Center for Genetic Engineering and Biotechnology, National Science and Technology Development Agency, Bangkok, Thailand; KU Leuven

## Abstract

We present RNA sequencing data sets and their genome sequence assembly for dengue virus that was isolated from a patient with dengue hemorrhagic fever and serially propagated in Vero cells. RNA sequencing data obtained from the first, third, and fifth passages and their corresponding whole-genome sequences are provided in this work.

## ANNOUNCEMENT

Dengue virus (DENV) belongs to the family *Flaviviridae*, genus *Flavivirus*. DENV consists of four serotypes, DENV1 to DENV4. All four serotypes are the cause of dengue fever (DF), dengue hemorrhagic fever (DHF), and dengue shock syndrome (DSS). DENV is an RNA virus that uses RNA-dependent RNA polymerase (RdRp) for replication. RdRp has poor proofreading capability, which results in high error rates during replication (1). Therefore, DENV populations rapidly mutate during propagation in the host cells. Here, we present DENV genome assemblies from raw reads of three serial passages of DENV in Vero cells (African green monkey kidney epithelial cells), which are commonly used for DENV isolation, titration, and propagation (2).

EDTA-treated plasma was collected in 2005 in southern Thailand from a patient with grade 2 DHF who was infected with DENV1. The patient was enrolled in a cohort study that had been approved by the ethical committee of the Faculty of Medicine Siriraj Hospital, Mahidol University, and the Ministry of Public Health in Thailand. The disease severity was classified according to the 1997 WHO guidelines (3). The DENV serotype was identified by reverse transcription-PCR (4). The virus was isolated by applying 150 μl of EDTA-treated plasma directly to the Vero cells. The inoculated Vero cells were maintained in growth medium for 5 days. On day 5, the supernatant was collected, centrifuged to remove cell debris, and aliquoted for storage as passage 1 (P1) samples. To start P2, 150 μl of P1 samples was inoculated in triplicate to fresh Vero cells. These processes were repeated to generate P3 to P5. The inoculations after virus isolation were performed in triplicate; therefore, three independent sets of samples were available for P2 to P5 but not for P1.

For DENV RNA sequencing, 1 ml of supernatant aliquot was centrifuged at 120,000 × *g* for 1.5 h at 4°C. The supernatant was carefully removed. The pellet was resuspended in 140 μl of 1× phosphate-buffered saline (5). The DENV RNA was extracted by using a QIAamp viral RNA minikit according to the manufacturer’s instructions. Samples from P1 (*n* = 1), P3 (*n* = 3), and P5 (*n* = 3) were selected for sequencing. The samples were sent to Macrogen (Seoul, South Korea) for the sequencing process. Briefly, each sample was used for library preparation with the TruSeq RNA sample preparation kit version 2. Then, each library was sequenced on the Illumina NovaSeq platform with 151-bp paired-end reads. The quality of the sequencing data was explored using FastQC version 0.11.8 (6). Low-quality bases (mean Phred quality score, <20) and adaptor sequences were trimmed from the end of each read using Trimmomatic version 0.36 (7). The whole-genome sequence of each sample was *de novo* assembled from cleaned sequencing data using IVA version 1.0.3 (8). The cleaned data were mapped to the corresponding assembled genome using BWA-MEM version 0.7.17 (9). All tools were run with default parameters. Assembly errors were explored by displaying the alignment result in the Integrative Genomics Viewer (IGV) tool version 2.4.14 (10, 11). The assembly errors in the form of substitutions were corrected, and indels were removed from the assembled genome sequences. The details of sequencing coverage, number of reads, GC content, and GenBank accession numbers are provided in Table 1. The coding regions of all seven genome sequences are identical except for the 5′ and 3′ untranslated regions, with the lengths varying from 86 to 94 bases and from 446 to 465 bases, respectively. These genome sequence data and raw read data for DENV serially cultured in Vero cells provide information on DENV evolution under selective pressure during continuous propagation in cell culture.

**TABLE 1:** Assembly metrics and accession numbers for each sample

Passage no.	Replicate no.	Sequence coverage (×)	GC content (%)	Total no. of reads	Genome coverage (nucleotide position)^*a*^	SRA accession no.	GenBank accession no.
1	1	277,861	46.84	26,313,496	1–10735	SRR12832840	MW362471
3	3.1	247,068	47.78	26,420,968	9–10735	SRR12832839	MW362472
	3.2	272,213	47.44	27,049,380	4–10728	SRR12832838	MW362473
	3.3	227,495	47.78	24,126,152	1–10735	SRR12832837	MW362474
5	5.1	194,806	46.98	24,527,494	9–10716	SRR12832836	MW362475
	5.2	132,483	46.16	21,663,268	9–10716	SRR12832835	MW362476
	5.3	146,317	47.97	30,241,236	1–10716	SRR12832834	MW362477

a Comparison between the assembled genome sequences and a DENV1 reference sequence from the GenBank database (accession number NC_001477).

### Data availability.

The RNA sequencing data for the seven samples were deposited in the Sequence Read Archive (SRA) database under the BioProject accession number PRJNA669806. The genome sequences were deposited in the GenBank database. The SRA and GenBank accession numbers are listed in Table 1.
